# Deciphering the role of signal regulatory protein α in immunotherapy for solid tumors

**DOI:** 10.3389/fimmu.2025.1612234

**Published:** 2025-06-16

**Authors:** Yulong Zhou, Xiyang Tang, Weiguang Du, Chen Shu, Xiaolong Yan, Nan Ma, Jinbo Zhao

**Affiliations:** ^1^ Department of Thoracic Surgery, Tangdu Hospital, The Fourth Military Medical University, Xi’an, Shaanxi, China; ^2^ Department of Cardiothoracic Surgery, The 902nd Hospital of the Chinese People’s Liberation Army Joint Logistic Support Force, Bengbu, Anhui, China; ^3^ Department of Ophthalmology, Tangdu Hospital, The Fourth Military Medical University, Xi’an, Shaanxi, China

**Keywords:** CD47, immunotherapy, immune checkpoint inhibitor, SIRPα, solid tumor

## Abstract

Therapies targeting immune checkpoints like programmed death receptor-1 and programmed death ligand-1 have demonstrated remarkable effectiveness in combating cancer. However, a subset of patients fails to respond to these therapies, underscoring the complexity of tumor immune evasion mechanisms. Exploring innovative immune regulatory targets represents a crucial research priority in this field. Signal regulatory protein α (SIRPα) is an immunosuppressive receptor expressed on myeloid cells that inhibits innate immunity through its interaction with the ligand integrin-associated protein (CD47). Blocking the SIRPα–CD47 axis can enhance myeloid cell-mediated anti-tumor responses and stimulate adaptive immunity, thereby synergizing with therapeutic antibodies and T-cell checkpoint inhibitors. Additionally, tumor-intrinsic SIRPα may facilitate tumor growth and immune evasion. This paper aims to elucidate the mechanisms of SIRPα activity in various cell types, review the advancements in SIRPα-targeted tumor therapies, and highlight the potential research value of tumor-expressed endogenous SIRPα.

## 1 Introduction

**Figure 1:**
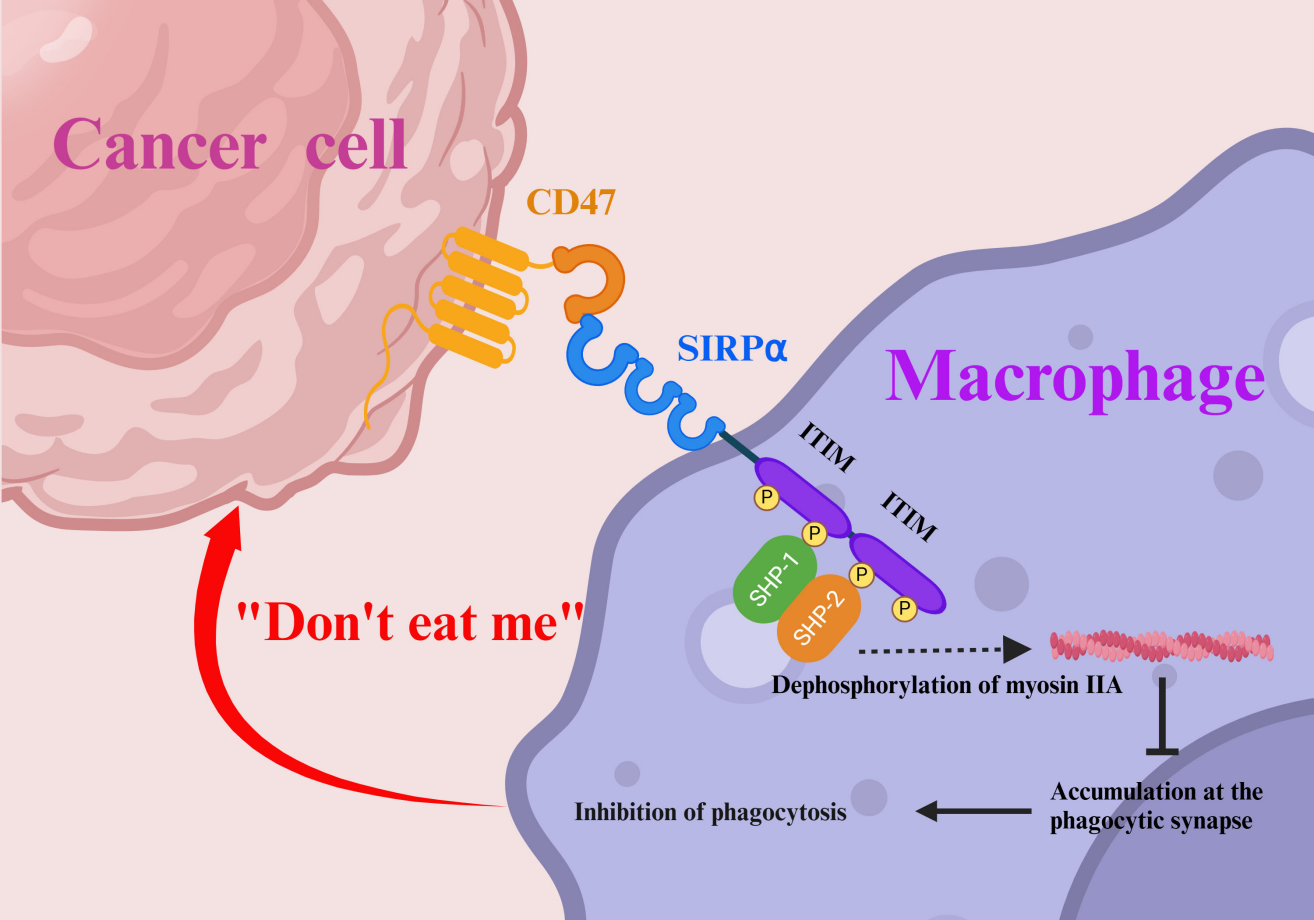
Tumor immune evasion via the “don’t eat me” signal. Cancer cells evade immune detection by exploiting the “don’t eat me” signal. The binding of SIRPα to CD47 initiates ITIM phosphorylation in the cytoplasm, recruiting SHP1 and SHP2 tyrosine phosphatases. This cascade dephosphorylates myosin IIA, preventing its accumulation at the phagocytic synapse and suppressing macrophage phagocytic signaling. SIRPα, signal regulatory protein α; ITIM, immunoreceptor tyrosine-based inhibitory motifs.

The clinical application of immune checkpoint inhibitors (ICIs) has profoundly transformed the landscape of cancer treatment (1). The majority of immune therapies activate adaptive immune responses that primarily target T-cell immune checkpoints (2). Programmed death receptor-1 (PD-1)/programmed death ligand-1 (PD-L1) inhibitors are currently the most widely used ICIs, with four anti-PD-1 and three anti-PD-L1 antibodies currently approved for clinical use (3, 4). The blockade of PD-1/PD-L1 can substantially slow down the progression of several solid tumors (5, 6). Despite satisfactory and lasting effects among responders, the therapeutic efficacy of these antibodies remains suboptimal for some patients. Therefore, more ICIs are yet necessary (7, 8). The immunosuppressive receptor known as signal regulatory protein α (SIRPα), expressed on myeloid cells, was developed and has received a lot of attention because of its function in mediating the immunosuppressive “don’t eat me” signal from cancer cells (9). It is widely recognized that cancer cells can upregulate integrin-associated protein (CD47) expression to exploit this “don’t eat me” signal to evade macrophage-mediated clearance and achieve immune evasion (**Figure 1**) (10). Studies have shown that targeting suppressive macrophages may enhance anticancer immune responses and improve the efficacy of immunotherapy combinations (11). Myeloid cells constitute a major component of the tumor microenvironment of solid cancers, whereas T-cell infiltration is often limited (12). The immunosuppressive cells within the tumor immune microenvironment inhibit T-cell activity through various mechanisms, thereby promoting cancer growth and metastasis (13, 14). Therefore, targeting myeloid cells within the tumor microenvironment, particularly through interventions aimed at their immune checkpoints, may offer novel strategies for inhibiting cancer progression. For example, blocking the CD47–SIRPα axis holds great potential as a novel immunotherapeutic approach (15). The structure and operation of SIRPα are covered in this review, along with a discussion of the molecular pathways by which SIRPα functions in various cells. We also present the research progress made toward anti-SIRPα antibody cancer therapies and discuss why a SIRPα-targeting strategy may be a valuable choice.

## 2 Structure and function of SIRPα

SIRPα is a member of the SIRP protein family, which comprises five distinct subtypes: SIRPα, SIRPβ1, SIRPβ2, SIRPγ, and SIRPδ. This protein, also referred to by various names such as CD172a, SHPS-1, p84, MFR, MYD-1, or PTPNS1, interacts exclusively with its ligand, CD47 (16, 17). SIRPα was expressed on myeloid cells, such as macrophages, neutrophils, dendritic cells, and microglial cells. It is also expressed at low levels in T-, B-, and natural killer (NK) cells (18). SIRPα is composed of three extracellular immunoglobulin superfamily domains. These domains include 1 variable and 2 constant type 1 domains. Additionally, SIRPα has one transmembrane region and an intracellular tail that can transmit inhibitory signals. Inside the intracellular tail, there are four tyrosine residues. These residues form two typical immunoreceptor tyrosine-based inhibitory motifs (ITIMs) (19, 20). Additionally, the extracellular immunoglobulin (Ig)V domain contains a ligand-binding region that allows SIRPα to interact with CD47, which consequently triggers a signaling cascade that can recruit the protein tyrosine phosphatases SHP1 and SHP2. This cascade results in the dephosphorylation of myosin IIA, which prevents its accumulation at the phagocytic synapse and ultimately leads to the suppression of phagocytic signals in macrophages, thereby protecting healthy cells from immune attacks. This inhibitory signal is known as the “don’t eat me” signal (21). Notably, the extracellular IgV domain of SIRPα is a hotspot for polymorphisms, with 10 human SIRPA alleles identified, the main variants being SIRPAV1, SIRPAV2, and SIRPAV8 (22–24). In turn, SIRPγ, which is primarily expressed on activated T-cells, has a much lower affinity for CD47 than that of SIRPα (25). Although the extracellular regions of SIRPγ and SIRPα share a high degree of homology (>70%), the intracellular domain of SIRPγ is notably shorter and fails to efficiently recruit signaling proteins, ultimately resulting in its lack of signaling potential. However, because of its binding ability to increase cell-cell adhesion, it can promote the production of synapses between T-cells and antigen-presenting cells (APCs), which increases the efficiency of antigen presentation and helps to mediate T-cell proliferation and cytokine secretion (26, 27). SIRPβ, expressed predominantly on myeloid cells, comprises 2 isoforms: SIRPβ1 and SIRPβ2. The SIRPβ2 isoform recruits the immunoreceptor tyrosine-based activation motifs-containing adaptor DAP12 via a transmembrane lysine residue to initiate immunostimulatory signaling, enhancing phagocytosis and antigen presentation by myeloid cells. Unlike SIRPα, SIRPβ2 does not interact with CD47, and its activation ligand remains unidentified. Similarly, while SIRPβ1 ligands are undefined, macrophage-specific SIRPβ1 engagement enhances phagocytic activity (28). Contrastingly, SIRPδ, a secreted isoform characterized by a single V-type Ig superfamily domain, is postulated to be expressed in spermatozoa and respiratory tissues (17).

## 3 Myeloid-intrinsic SIRPα regulates the tumor immune microenvironment

### 3.1 Functional role of SIRPα in macrophages

**Figure 2:**
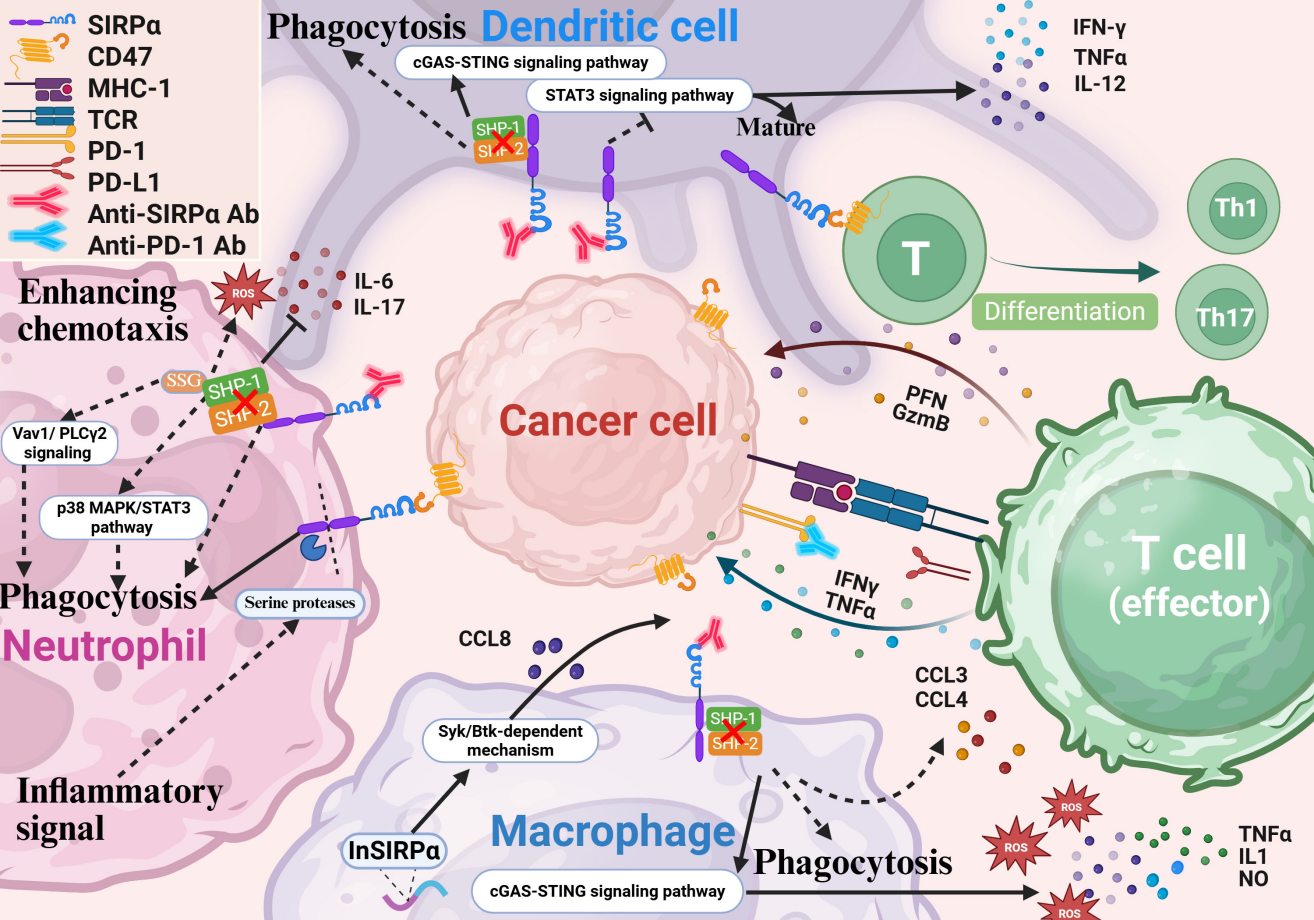
SIRPα blockade enhances innate and adaptive immunity. Inhibition of SIRPα boosts the phagocytic and antigen-presenting capabilities of myeloid cells. In macrophages, SIRPα blockade activates inflammatory pathways and the cGAS-STING cascade in CAR macrophages, increasing the secretion of IL-1, TNF-α, ROS, nitric oxide, and chemokines like CCL3 and CCL4. It also promotes T-cell recruitment in tumors through a Syk-Btk-dependent mechanism. In neutrophils, SIRPα inhibition enhances chemotaxis, infiltration, and cytotoxicity. During inflammation, neutrophil ITIM cleavage generates a truncated receptor that binds CD47 without transmitting inhibitory signals, further enhancing chemotaxis, ROS release, and phagocytosis. In DCs, SIRPα blockade suppresses STAT3 signaling, increases cytokine secretion (e.g., IL-12, TNF-α, and IFN-γ), and promotes DC maturation. It also activates cGAS-STING signaling, improving tumor antigen cross-presentation. Additionally, SIRPα on DCs modulates naive T-cell differentiation into helper T-cells. SIRPα, signal regulatory protein α; CAR, chimeric antigen receptor; DC, dendritic cell; IL, interleukin; TNF, tumor necrosis factor; INF, interferon.

Blocking SIRPα can enhance antibody-dependent cellular phagocytosis (ADCP) by macrophages, features that have attracted significant attention for research (29–31) (**Figure 2**). Microglia play a similar functional role to macrophages in central nervous system tumors. They function as the effector cells in the disruption of the CD47-SIRPα anti-phagocytic axis (32, 33). Generally, promoting ADCP is achieved by blocking the binding of SIRPα to CD47 to abolish the “don’t eat me” signal. Furthermore, in chimeric antigen receptor macrophages, SIRPα inhibition in macrophages can activate inflammatory pathways and the cGAS–STING signaling cascade, leading to an elevated production of proinflammatory cytokines, such as interleukin-1 (IL-1), tumor necrosis factor-alpha (TNF-α), reactive oxygen species (ROS), and nitric oxide, which increase the anticancer activity (34, 35). Moreover, preventing the expression of SIRPα in macrophages induces the recruitment and migration of T-cells via increased secretion of chemokines (e.g., C-C motif chemokine ligands CCL3 and CCL4) (26). In SIRPα-knockout (SIRPα-KO) mice, SIRPα-KO macrophages were found to display robust anticancer activity and antigen-presenting capacity, which was associated with enhanced T-cell activation and proliferation. Notably, SIRPα-KO macrophages were found to promote T-cell recruitment in cancers via a Syk–Btk-dependent mechanism involving CCL8 secretion, transforming tumor-associated macrophages and granulocytic myeloid-derived suppressor cells into subsets expressing high levels of CCL8 and H2-Q10, respectively, with enhanced antigen presentation, phagocytosis, inflammatory response, and chemotaxis capacities (36). Therefore, targeting SIRPα has the potential to reprogram the tumor immune microenvironment, promoting systemic anticancer responses and preventing solid cancer progression. In brief, by blocking the expression of SIRPα in macrophages, the traditional “don’t eat me” signaling pathway can be suppressed, which will improve phagocytosis and stimulate macrophages to secrete chemokines and cytokines via additional signaling pathways. Simultaneously, it has the potential to block the SIRPα-mediated non-CD47-dependent pathway, reprogramming the suppressive tumor immune microenvironment.

### 3.2 Functional role of SIRPα in neutrophils

In cancer therapeutics, anti-SIRPα antibodies exert their antitumor effects by disrupting the CD47-SIRPα interaction and relieving inhibitory signaling on neutrophils.) (37). When combined with tumor-targeting antibodies, the Fc region of these therapeutic antibodies engages activating Fcγ receptors (e.g., FcγRIIIa) on neutrophils, triggering antibody dependent cell-mediated cytotoxicity (ADCC) and subsequently enhancing neutrophil-mediated tumor cell killing (3, 38). Sodium stibogluconate (SSG), a selective SHP-1 inhibitor, enhances neutrophil cytotoxicity by blocking phosphatase-mediated suppression of Vav1 and PLCγ2 signaling. Co-administration of SSG with CD47-SIRPα blockade amplifies ADCC efficacy through dual inhibition of immunosuppressive pathways (39). SIRPα signaling suppresses neutrophil phagocytic activity and cytotoxicity through the SHP-1/p38 MAPK/STAT3 pathway while promoting IL-6 and IL-17 secretion. After SIRPα-KO, neutrophils polarize toward the anti-tumor N1 phenotype, with enhanced phagocytic function and reduced inflammatory cytokine secretion, thereby inhibiting the growth of lung cancer (40). However, compared with IgA, IgG-mediated ADCC exhibits relatively low efficiency (41–45). Blocking SIRPα on neutrophils with anti-SIRPα antibodies significantly enhances ADCC mediated by IgA2 variants of cetuximab and trastuzumab against HER2-positive breast cancer cells and EGFR-positive epidermoid carcinoma cells (46). Paradoxically, SIRPα overexpression in autoimmune lesions (e.g., rheumatoid arthritis and inflammatory bowel disease) exacerbates inflammation through dysregulated innate immunity (47). During chronic inflammation, neutrophil-derived serine proteases cleave the SIRPα ITIM domain in an IL-17-dependent manner. The resultant truncated SIRPα retains CD47-binding capacity but loses inhibitory signaling, unleashing neutrophil chemotaxis, ROS production, and phagocytic activity (48).

### 3.3 Functional role of SIRPα in dendritic cells

As specialized APCs, dendritic cells (DCs) are crucial in facilitating T-cell activation and maintaining immune tolerance (49, 50). When DCs come into contact with cancer cells, they send a “don’t eat me” signal through the classic ITIM–SHP1 complex that mediates anti-phagocytic effects but also through SIRPα, which detects cancer mitochondrial DNA for cross-priming or activate the STAT3 signaling pathway to suppress the production of cytokines (such as IL-12, TNF-α, and interferon-γ) and consequently inhibit DC maturation. Additionally, the PI3K–AKT signaling pathway also plays a pivotal role in regulating the activation and maturation of DCs through SIRPα (51, 52). Combined therapy with radiotherapy/anti-SIRPα/anti-PD-1 for colorectal cancer was shown to effectively induce cGAS–STING signaling in DCs both *in vitro* and *in vivo*, facilitating efficient cross-presentation of tumor-associated antigens (53, 54). Moreover, when SIRPα was silenced in DCs, increased secretion of cytokines (e.g., TNF-α, IL-12, and IL-6), enhanced the secretion of interferon-γ by CD8+ T lymphocytes, and effectively killed cervical cancer cells *in vitro (* 55). Of note, the interaction between SIRPα on DCs and CD47 on T-cells modulates the differentiation of naïve T-cells into T-helper (Th) cells. Mice lacking SIRPα exhibit enhanced resistance to autoimmune diseases caused by Th1 or Th17 cells, such as encephalomyelitis and colitis (56–59). Besides regulating T-cells by presenting tumor antigens, SIRPα can further influence the differentiation and function of T-cells by regulating their own maturation. Thus, blocking SIRPα can promote DC maturation and enhance their antigen-presenting function, thereby facilitating the function of cytotoxic T-cells.

## 4 Role of tumor-intrinsic SIRPα in tumor progression

**Figure 3:**
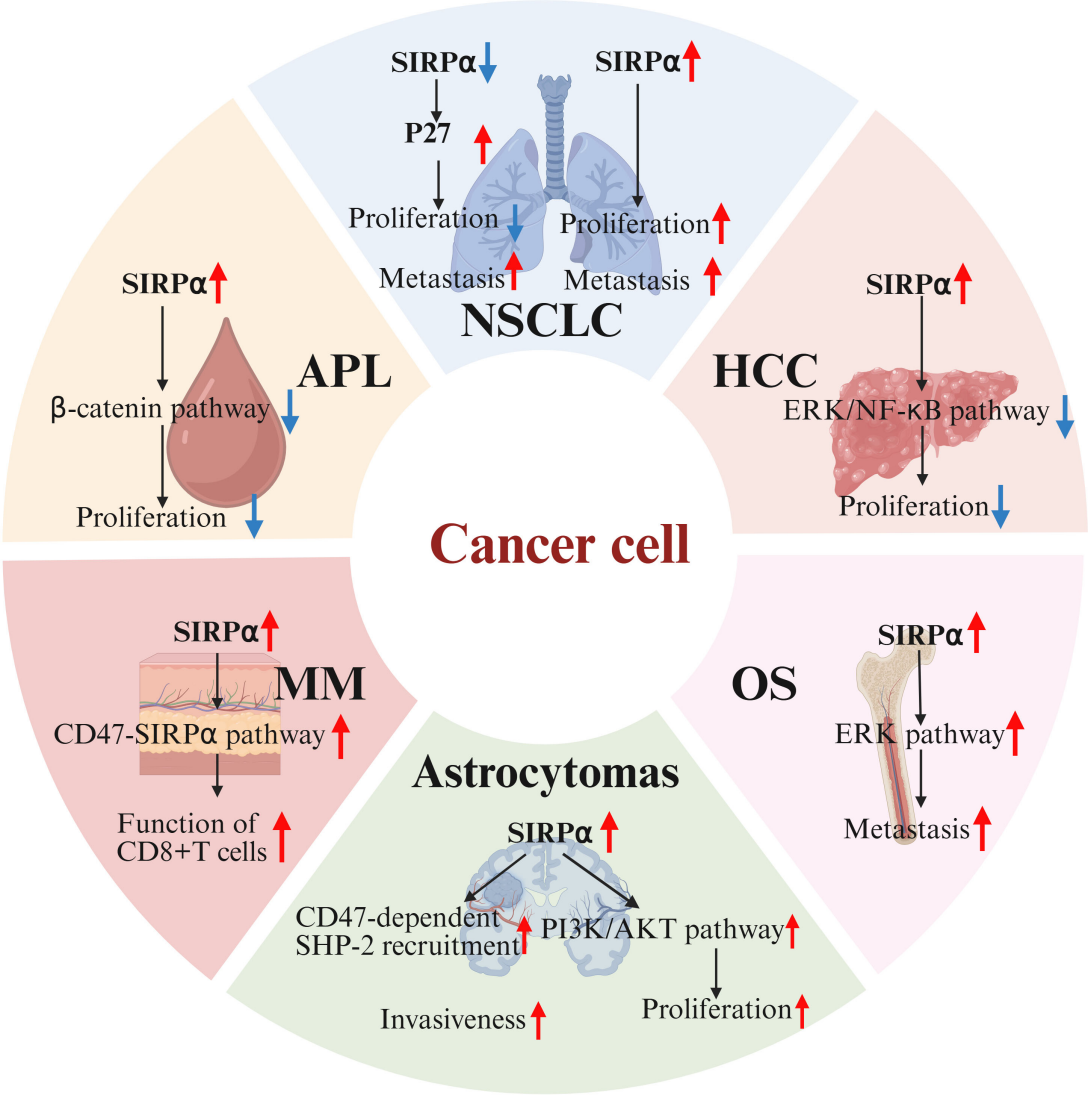
Endogenous SIRPα in tumor malignancy. In osteosarcoma(OS), SIRPα overexpression activates the ERK pathway, driving metastasis. In acute promyelocytic leukemia(APL), SIRPα upregulation may inhibit β-catenin signaling, suppressing tumor proliferation. In hepatocellular carcinoma(HCC), SIRPα negatively regulates tumor initiation by inhibiting ERK and NF-κB pathways. In non-small cell lung cancer(NSCLC), SIRPα knockdown upregulates p27, reducing tumor growth, but p27 mislocalization to the cytoplasm paradoxically increases invasiveness. Conversely, SIRPα overexpression enhances cell proliferation and migration. In melanoma(MM), SIRPα overexpression augments CD8+ T-cell function. In astrocytomas, SIRPα may be involved in cell adhesion and signal transduction through CD47-dependent phosphorylation and SHP-2 recruitment, thereby affecting tumor invasiveness. Additionally, SIRPα may regulate tumor proliferation and survival by inhibiting growth factor signaling or by modulating the PI3K/AKT pathway. SIRPα, signal regulatory protein α; ERK, extracellular signal-regulated kinase.

In summary, targeting the immune checkpoint receptor SIRPα can boost both innate and adaptive immune responses, offering novel strategies for cancer immunotherapy. Surprisingly, some solid cancers (such as renal cell carcinoma, colorectal cancer, and osteosarcoma) exhibit high levels of SIRPα expression. Despite the limited research on endogenous SIRPα in cancer cells, multiple pivotal studies have shed light on the role of endogenous SIRPα in the malignant progression of cancers (**Figure 3**) (60, 61). Specifically, in osteosarcoma cells, the upregulation of SIRPα activates the extracellular signal-regulated kinase (ERK) pathway, leading to the phosphorylation of specificity protein 1 (Sp1) at the threonine 278 site. This phosphorylated protein then binds to the promoter region of solute carrier family 7 member 3 (SLC7A3), resulting in increased SLC7A3 expression and enhanced cellular arginine uptake capacity. These processes collectively promote the metastasis of osteosarcoma (62). Contrastingly, in acute promyelocytic leukemia (APL) cells, overexpression of SIRPα exhibits distinct effects, potentially inhibiting the β-catenin signaling pathway and upregulating Foxo3a expression, which in turn induces apoptosis and inhibits tumor cell proliferation (63). In hepatocellular carcinoma cells, SIRPα has been shown to negatively regulate tumor initiation, primarily through the inhibition of the ERK and NF-κB pathways (64). Similarly, SIRPα is used by non-small cell lung cancer as a critical regulator of the EGFR pathway. Knockdown of SIRPα induces the upregulation of p27, subsequently inhibiting cell cycle progression and reducing tumor growth. However, increased p27 expression leads to its mislocalization to the cytoplasm, paradoxically promoting cancer cell invasiveness. Conversely, the enhanced expression of SIRPα boosts the cell’s migratory and proliferative capabilities. These findings suggest that SIRPα may exert dual oncogenic or tumor-suppressive properties, depending on its regulation of multiple signaling pathways within cancer cells (65). Additionally, Z. Zhou and his research team uncovered a unique function of SIRPα in melanoma cells: as a marker for melanoma cells, the expression level of SIRPα diminishes progressively as melanoma progresses. SIRPα interacts with CD47, modulating the function of CD8+ T-cells. Studies have shown that cytotoxic T-cells exert stronger anti-melanoma effects on cells overexpressing SIRPα, and the addition of anti-PD-L1 antibodies significantly enhances this killing effect. This suggests that endogenous SIRPα in melanoma cells plays a positive role in PD-1/PD-L1-induced T-cell-mediated anticancer immunity, while the absence of SIRPα may lead to increased resistance to PD-L1 therapy (66). Microglia critically shape developing neural circuits by eliminating redundant synapses via phagocytic activity. Genetic ablation of neuronal SIRPα suppressed microglial synaptic engulfment, resulting in elevated retinal synapse density. Conversely, sustained neuronal SIRPα expression prolonged phagocytic activity and decreased synaptic numbers. Mechanistically, neuronal SIRPα serves as a decoy receptor that sequesters inhibitory CD47 signals from microglial SIRPα, thereby enabling synapse clearance. This SIRPα-CD47 regulatory axis elucidates a molecular basis for pathological synapse loss in neurological conditions (67). SIRPα is not expressed in normal astrocytes but exhibits functional expression in astrocytomas, potentially participating in cell adhesion and signaling through CD47-dependent phosphorylation and SHP-2 recruitment, thereby influencing tumor invasiveness. Furthermore, SIRPα may regulate tumor proliferation and survival by either suppressing growth factor signaling or modulating the PI3K/AKT pathway. Its potential as a therapeutic target or prognostic biomarker in astrocytomas warrants further investigation (68).

## 5 Advances in therapeutic targeting of SIRPα in solid tumors

### 5.1 Preclinical studies on anti-SIRPα therapy in solid tumors

Multiple anti-SIRPα antibodies developed for solid tumor treatment in preclinical studies have demonstrated significant efficacy in suppressing tumor progression (38, 69) (**Table 1**). Yanagita et al. validated the tumor-inhibitory effect of the mouse-derived anti-SIRPα monoclonal antibody MY-1, which showed enhanced cytotoxicity against HER2-positive breast cancer cells *in vitro*. It significantly inhibited the growth of SIRPα-expressing renal cell carcinoma and melanoma cells, but not of non-SIRPα-expressing cells. Combination with rituximab or anti-PD-1 antibody further enhanced the ability of MY-1 to suppress the growth of Burkitt lymphoma and colorectal cancer cells. Moreover, when used as a monotherapy, MY-1’s anticancer activity against renal cell carcinoma and melanoma was mediated by macrophages, but also NK and CD8+ T -cells (60). In SIRPα-deficient mice, MY-1 monotherapy showed inhibition of cancer growth by binding to SIRPβ and promoting ADCP (70). The effects of MY-1 differ between tumors with and without SIRPα expression, indicating that endogenous SIRPα in cancer cells is involved in certain regulatory mechanisms.

**Table 1:** Preclinical characteristics of anti-SIRPα antibodies in solid tumors.

Antibody	Mechanism of Action	Monotherapy Efficacy	Combination Therapy	Safety Profile
MY-1	Blocks SIRPα; activates macrophages, NK cells, and CD8+ T cells; binds SIRPβ in SIRPα-deficient mice	Suppresses HER2+ breast cancer (*in vitro*), renal cell carcinoma, and melanoma (*in vivo*)	Synergizes with rituximab/anti-PD-1 (Burkitt lymphoma, colorectal cancer)	No severe toxicity reported; macrophage-dependent activity
KWAR23	Binds SIRPγ; no direct phagocytosis induction	Limited efficacy as monotherapy	Enhances T-cell function; no immune cell infiltration in brain tissue	No neurological abnormalities observed
SIRP-1	Blocks SIRPα; induces internalization of SIRPα/antibody complex	Phagocytosis dependent on macrophage CD32 (FcγRII)	Not explicitly reported	Reduces macrophage SIRPα levels
SIRP-2	Alters SIRPα-CD47 affinity via dimerization modulation	Similar to SIRP-1	Not explicitly reported	Modulates macrophage SIRPα aggregation
BR105	Pan-allele binder; mild SIRPγ binding	Ineffective as monotherapy	Not explicitly reported	Well-tolerated in non-human primates; no adverse reactions
1H9	Blocks SIRPα; limited antigen sink effect	Inhibits tumor progression without T-cell interference	Superior to anti-CD47 in CD47/SIRPα double-humanized mice	Reduced antigen sink effect; enhanced biosafety
hAB21	Competes with cetuximab for FcγR binding (“scorpion effect”)	Limited phagocytosis enhancement	Synergizes with anti-PD-1/PD-L1; no anemia in cynomolgus monkeys	Safe in primates; avoids FcγR competition
CTX-5861	Bispecific (SIRPα + PD-L1); enhances phagocytosis and antigen presentation	Not explicitly reported	Dual targeting improves macrophage and dendritic cell activity	Designed to minimize off-target effects
AL008	Triggers SIRPα degradation; activates FcγR via Fc domain	Monotherapy efficacy in triggering myeloid activation	Enhances anti-PD-L1 activity	Pan-allele coverage; no reported toxicity

Humanized SIRPα antibodies can effectively block various human SIRPα variants. Several antibody monotherapies each have their own characteristics. KWAR23 alone fails to induce macrophage phagocytosis. Moreover, no immune cell infiltration or obvious neurological abnormalities were observed in the brains of mice treated with KWAR23; however, it binds to SIRPγ and affects T-cell function (38). The phagocytic activity of SIRP-1 and -2 is important as monotherapy depends on the “eat me” receptor CD32 (FcγRII) in macrophages. SIRP-1 functions by directly blocking SIRPα and inducing internalization of the SIRPα/antibody complex, thereby reducing the levels of SIRPα in macrophages, while SIRP-2 alters the affinity of SIRPα for CD47 by affecting its dimerization/aggregation in macrophages (69). BR105 is ineffective when used alone; although it can mildly bind to SIRPγ, it does not inhibit T-cell activation. Toxicity studies in non-human primates showed that BR105 is well-tolerated, with no treatment-related adverse reactions observed (71). 1H9 exhibits a similar effect in inhibiting cancer progression without affecting T-cell function. When comparing anti-SIRPα and anti-CD47 antibodies using CD47/SIRPα double-humanized mice, it was found that 1H9 exhibits significantly reduced antigen sink effect and enhanced biosafety owing to the limited tissue distribution of SIRPα expression (72).

When combined with therapeutic antibodies, such as rituximab, all antibodies demonstrate significant inhibitory effects on the growth of hematological malignancies and solid cancers both *in vitro* and *in vivo*. Additionally, several antibodies when used in combination with ICIs exhibit good safety and therapeutic effects. Competition between hAB21 and cetuximab for macrophage FcγR limits the ability of anti-SIRPα antibodies to enhance macrophage phagocytosis. Alternatively, hAB21 with an active Fc structure can co-bind to SIRPα and FcγR in macrophages, leading to heterotrimeric interactions that restrict the binding of cetuximab to macrophage FcγR, thereby reducing phagocytic signaling. This phenomenon is known as the “scorpion effect.” When combined with anti-PD-1 or anti-PD-L1 antibody blockade therapy, hAB21 significantly inhibits the growth of tumor cells and does not cause anemia or other adverse outcomes when used in cynomolgus monkeys (11). CTX-5861 is a bispecific antibody targeting both SIRPα and PD-L1, designed to enhance macrophage phagocytosis and improve the efficiency of antigen presentation by DCs (73). AL008, a specific antibody targeting pan-alleles of SIRPα, demonstrates monotherapy efficacy by triggering SIRPα degradation and stimulating the activation of FcγR on bone marrow cells via its Fc domain. Additionally, the antitumor activity of anti-PD-L1 drugs has also been enhanced (74).

### 5.2 Clinical studies on anti-SIRPα therapy in solid tumors

Although the aforementioned anti-SIRPα antibodies have not entered the clinical trial stage, some anti-SIRPα antibodies have demonstrated good biosafety and cancer treatment efficacy in preclinical studies and have thus entered clinical trials (**Table 2**). ADU-1805, a humanized IgG2 anti-SIRPα antibody, does not affect T-cell activation or bind to red blood cells/platelets. In non-human primates, ADU-1805 exhibited no toxicity. Furthermore, ADU-1805 does not bind to macrophage FcγRIIA to trigger the “scorpion effect,” nor does it induce NK cell-mediated ADCC, lacks activity in mediating complement-dependent cytotoxicity, and does not stimulate cytokine secretion in human whole blood, further substantiating its clinical viability. ADU-1805 is undergoing clinical trials (NCT05856981), and the results are yet to be announced (27, 75). BI 765063 is a humanized IgG4 monoclonal antibody antagonist of SIRPα that binds with high affinity to SIRPαV1 but not to SIRPγ, thereby preserving T-cell function. Ongoing research (NCT05249426) to test whether different combinations of BI 765063, Ezabenlimab, chemotherapy, cetuximab and BI 836880 are helpful for patients with head and neck or liver cancer. Another clinical trial (NCT03990233) is currently evaluating the safety and efficacy of BI 765063 as monotherapy or in combination with ezabenlimab in patients with advanced solid tumors. BI 765063 monotherapy was found to be well-tolerated and showed activity, with treatment biopsies from responders demonstrating increased CD8+ T-cell infiltration and activation (76). Additionally, a clinical trial in Japan (NCT04653142) assessed the safe dose of BI 765063 in Japanese patients and found that its safety and pharmacokinetic parameters were consistent with those observed in Caucasian patients (77). A study (NCT05446129) aimed at evaluating the safety, feasibility, efficacy, and biological activity of the neoadjuvant treatment with Ezabenlimab combined with BI 765063 and pembrolizumab combined with BI 765063 in newly diagnosed patients with locally regional colorectal cancer has been dropped by the pharmaceutical company. BI 770371 is a pan-specific monoclonal antibody against SIRPα currently being evaluated the tolerability of different doses of BI 770371 when used alone or in combination with ezabbenlimab (NCT05327946). It is considered that the toxicity profile of BI 770371, both as a monotherapy and in combination therapy, is manageable. Another study (NCT05068102) aimed at finding out how the two drugs, BI 765063 and BI 770371, are absorbed in tumors and how they are distributed in the body is underway (78). CC-95251(BMS-986351) is a fully human monoclonal antibody targeting SIRPα, with preclinical studies showing its ability to enhance macrophage phagocytic activity when combined with the therapeutic antibody rituximab (79). A clinical trial (NCT03783403) is evaluating CC-95251 as a monotherapy and in combination with cetuximab and rituximab for safety, tolerability, and preliminary clinical activity in participants with advanced solid and hematological malignancies. Unfortunately, the clinical trial has been terminated owing to changes in business objectives (80). DS-1103a, a recombinant humanized IgG4 antibody targeting SIRPα, is currently being assessed in combination with T-DXd for its efficacy, recommended dosage, and pharmacokinetic properties in patients with advanced solid tumors (NCT05765851). IBI397, a pan-allelic antibody against SIRPα, underwent clinical trials for advanced malignant tumors but the trial (NCT05245916) was withdrawn owing to changes in the company’s development strategy.

**Table 2:** Various anti-SIRPα antibodies are involved in multiple clinical trials.

First Submitted	Drug names	Categories	Clinical Trials	Indications	Phase	Clinical Status
2023/1/4	ADU-1805	An anti-SIRPα pan-allelic humanized monoclonal IgG2 antibody	NCT05856981	Advanced Solid Cancers	1	Recruiting
2019/5/21	BI 765063	An anti-SIRPα V1 variant IgG4Pro antibody	NCT03990233	Advanced Solid Cancers	1	Active
2020/11/27			NCT04653142	Advanced Solid Cancers	1	Completed
2022/2/10			NCT05249426	Head and Neck Cancer or Liver Cancer	1	Active
2022/7/1			NCT05446129	Colorectal Cancer	1	Terminated
2021/9/19	BI 770371	An anti-SIRPα V1 and V2 variant IgG1 antibody	NCT05068102	Advanced Head and Neck Cancer, Skin Cancer, or NSCLC	1	Recruiting
2022/4/8			NCT05327946	Advanced Solid Cancers	1	Active
2018/12/19	CC-95251 (BMS-986351)	An anti-SIRPα humanized monoclonal antibody	NCT03783403	Advanced Solid and Hematologic Cancers	1	Terminated
2023/3/1	DS-1103a	An anti-SIRPα humanized IgG4 antibody	NCT05765851	Advanced Solid Cancers	1	Recruiting
2022/1/26	IBI397	An anti-SIRPα pan-allelic antibody	NCT05245916	Advanced Malignancies	1	Withdrawn

## 6 Why select an anti-SIRPα antibody therapeutic strategy?

### 6.1 Limitations of CD47-targeted therapy in solid tumors

Current developments of CD47–SIRPα signaling pathway inhibitors can be roughly categorized into three types: (i) blockers of CD47 molecules in target cells, which includes anti-CD47 antibodies and SIRPα-Fc fusion antibodies, (ii) blockers of SIRPα molecules in immune effector cells, and (iii) inhibitors of glutaminase-like proteases (81). Anti-CD47 antibodies have been shown to achieve objective (total or partial) remission in 50% of patients by showing considerable anticancer activity in hematological malignancies. However, treatment of solid cancers has led to adverse effects, including anemia (57% of patients) and lymphocytopenia (34% of patients) (82–84). Although strong effects in preclinical studies were observed, especially those that retain large Fc receptor (FcR) inactivation potential in human IgG1 molecules, their clinical value may be limited by non-tumor toxicity (18). The primary reason is that CD47 lacks cancer specificity and is widely distributed in healthy tissues, leading to a substantial “antigen sink”; thus, high doses of anti-CD47 drugs are required to attain anticancer efficacy. Moreover, many anti-CD47 antibodies retain effector functions via their immunoglobulin Fc domains, which may trigger macrophages to engage in ADCP against healthy cells (85–87). Indeed, anemia and thrombocytopenia are common side effects of such anti-CD47 antibodies, often requiring red blood cell transfusions and low-dose initiation strategies to mitigate the adverse situation (82, 88). To manage these risks, current research on anti-CD47 antibodies is focused on molecules that reduce FcγR binding ability, such as IgG4 antibodies. Most of these molecules can still induce severe anemia in non-human primates and cancer patients. Moreover, anti-CD47 antibodies may affect how much CD47 interacts with other receptors, such as integrins, vascular endothelial growth factor receptor-2 (89), thrombospondin-1 (90), and SIRPγ (91). Notably, blocking the interaction between CD47 and SIRPγ can inhibit T-cell extravasation and activation, thereby diminishing the anticancer response. Hence, CD47 signals appear to have a more complex biological functions and its blockade may elicit unexpected cellular responses (3, 27). Additionally, the anticancer activity of anti-CD47 antibodies depends on CS1 glycoprotein antigen (SLAMF7) phagocytic signaling, which is generally absent in solid cancers but is expressed in hematological malignancies (86, 92). Since 2022, multiple Phase III clinical trials of magrolimab were terminated or suspended owing to a lack of survival benefits or adverse reactions, with the regulatory agency also pausing some clinical studies of magrolimab in solid cancers (93). The side effects caused by non-targeted cancer cells and the negative impact on the interaction between CD47 and other receptors have become major obstacles limiting the widespread application of first-type antibodies in the treatment of solid cancers (94).

### 6.2 Prospects of therapeutic targeting of SIRPα in solid tumors

SIRPα is predominantly expressed in myeloid cells, including monocytes, granulocytes, DCs, macrophages, and microglia, which demonstrates a more limited histological distribution than CD47. SIRPα blocking agents are less likely to be influenced by constraints on antigen expression. Therefore, therapies targeting SIRPα have the potential to avoid side effects associated with targeting CD47 (95, 96) (**Table 3**). Research on anti-SIRPα antibodies indicates that, similar to CD47 blocking antibodies devoid of Fc, SIRPα blocking agents lacking Fc can effectively induce anticancer immune responses when used along with T-cell-targeted therapies (11). Moreover, monotherapy with anti-SIRPα can alter the composition of the immune cell population in the tumor microenvironment, as evidenced by a significant increase in the proportion of M1 macrophages and a decrease in M2 macrophages (60, 97). SIRPα can negatively regulate DC activation and maturation, thus inhibiting SIRPα can enhance DC responses (52). Anti-SIRPα antibody therapy can stimulate an influx of tumor-infiltrating NK cells and CD8+ T-cells, as well as induce DC activation and promote T-cell effector function when used in combination with anti-PD-1 antibodies (36). The blockade of the SIRPα–CD47 signaling pathway combined with T-cell ICIs can enhance adaptive immune responses. Although the strategy of inhibiting SIRPα has advantages, such as increased antitumor responses and lack of red blood cell toxicity, the high polymorphism rate of the distal IgV domain in the extracellular region of SIRPα raises a risk of cross-reactivity with other members of the SIRP family. This makes the development of clinically beneficial SIRPα inhibitors particularly challenging (27).When glutaminase-like proteases are inhibited, newly synthesized CD47 molecules are unable to effectively bind to their natural binding partners owing to the lack of pyroglutamate modification. Unlike antagonistic molecules targeting CD47 or SIRPα directly, small-molecule inhibitors for this pathway do not compete with natural binding partners in the tumor microenvironment. Moreover, small-molecule inhibitors have high tissue penetration and potential oral bioavailability, which makes them an attractive option. However, the risk of blocking other functions of CD47 persists with small-molecule inhibitors (81). Collectively, anti-SIRPα antibodies capable of blocking all SIRPα alleles hold promise as competitive candidates for achieving the clinical goal of halting the progression of solid cancers.

**Table 3:** The pros and cons of anti-CD47 antibodies versus anti-SIRPα antibodies.

Antibody Type	Anti-CD47 Antibody	Anti-SIRPα Antibody
Target Distribution	Broadly expressed in normal cells (e.g., red blood cells)	Expressed exclusively in myeloid cells (e.g., macrophages, dendritic cells)
Biosafety	High hematotoxicity risk (anemia, thrombocytopenia)	Favorable safety profile; low hematotoxicity risk
Mechanism of Action	Blocks CD47-SIRPα signaling; activates macrophages via Fc-dependent mechanisms	Blocks CD47-SIRPα signaling; directly engages FcγR to activate macrophages
Therapeutic Potential	More effective against hematologic malignancies	Effective in solid tumors
Clinical Maturity	Multiple agents in late-stage trials (e.g., Magrolimab)	Majority in early-stage clinical development
Clinical Challenges	Requires antibody engineering to mitigate hematotoxicity	Develop broad-spectrum antibodies to target SIRPα pan-alleles

## 7 Discussion

**Figure 4:**
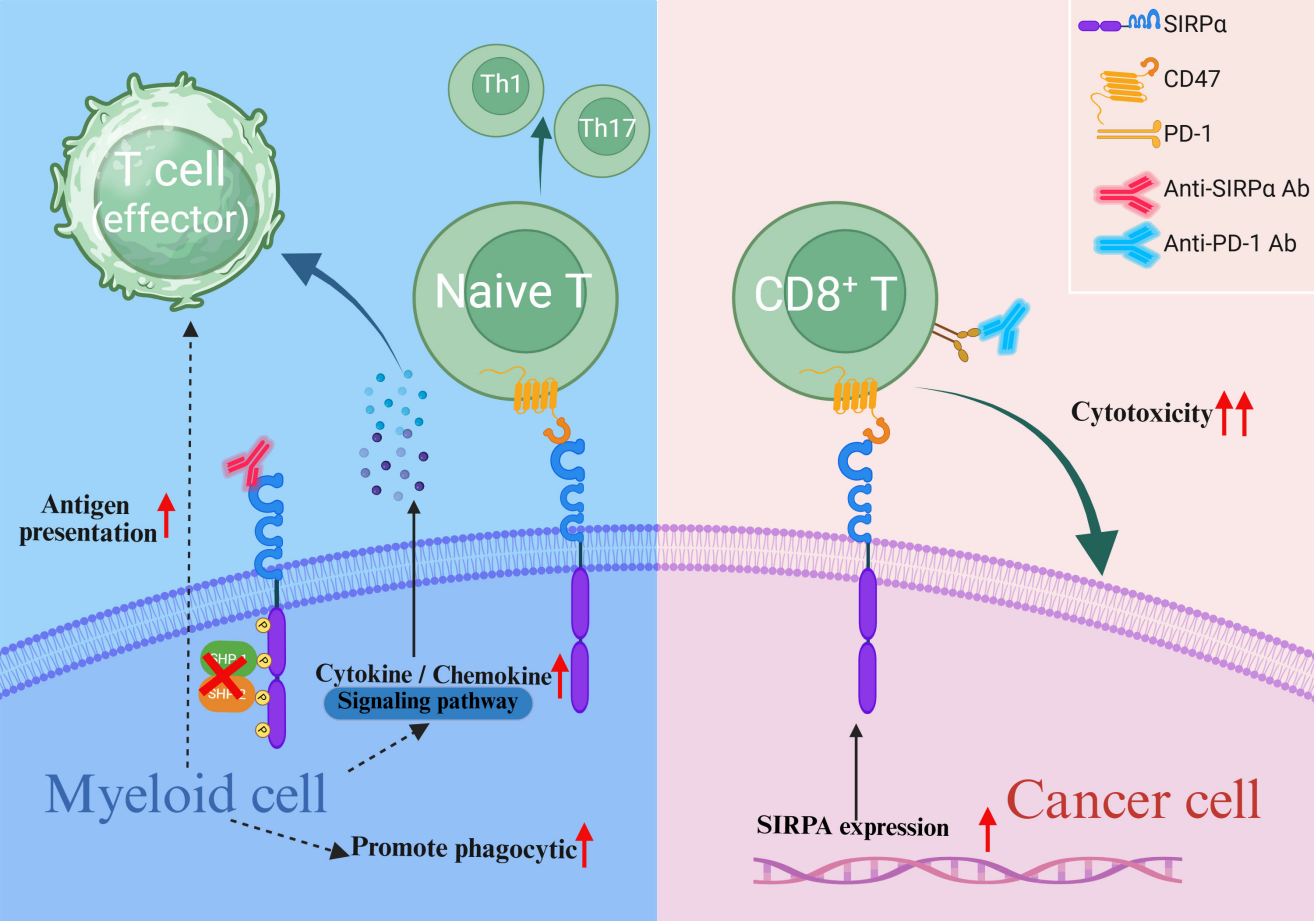
Endogenous SIRPα in tumor malignancy. Blockade of myeloid-intrinsic SIRPα enhances the antigen-presenting capacity of myeloid cells, promotes the release of pro-inflammatory cytokines and chemokines, augments the cytotoxic activity of CD8+ T cells, and modulates the differentiation of T helper cells. Elevated tumor-intrinsic SIRPα expression may also potentiate CD8+ T cell cytotoxicity.

The CD47–SIRPα interaction plays a key regulatory role in numerous biological processes that influence cellular fate. It is not only viewed as a highly promising target in the field of cancer immunotherapy, but also holds significant importance for maintaining physiological tissue homeostasis (25, 98–100). Collectively, myeloid-intrinsic SIRPα modulates the tumor immune microenvironment by regulating the immunomodulatory functions of macrophages, neutrophils, and DCs. Contrastingly, tumor-intrinsic SIRPα primarily influences malignant phenotypes—such as proliferation, migration, and invasion—via direct intracellular signaling pathways(**Figure 4**). Notably, although T-cells do not express SIRPα, macrophages and DCs exert multifaceted regulation over T-cell functionality through SIRPα-dependent mechanisms. Blockade of SIRPα enhances antigen presentation in macrophages, promotes the release of pro-inflammatory cytokines, and recruits T-cells to remodel the immunosuppressive tumor microenvironment. Similarly, SIRPα inhibition in DCs alleviates its suppressive effects on antigen presentation, activates cGAS-STING signaling, and stimulates cytokine secretion, directly augmenting CD8+ T-cell cytotoxicity while balancing Th cell differentiation to optimize immune responses. Intriguingly, tumor-intrinsic SIRPα expression may also regulate T cell function. For instance, melanoma cells with low SIRPα expression exhibit suppressed CD8+ T-cell cytotoxicity. However, the molecular mechanisms by which tumor-intrinsic SIRPα modulates T-cell activity remain poorly characterized. These insights underscore the multifaceted role of SIRPα in the tumor ecosystem. Researchers have developed various humanized anti-SIRPα antibodies that have shown excellent anticancer effects in preclinical studies, and some of these antibodies have entered clinical trials. Although the unique pharmacokinetics and biosafety of anti-SIRPα antibodies are highly anticipated, the high polymorphism rate of the human SIRPα V domain poses a challenge for the development of SIRPα-targeting drugs. Fortunately, three allelic combinations (V1/V1, V1/V2, and V2/V2) cover almost the entire human population. In addition to the development of humanized pan-allele-targeting antibodies, future research should focus on designing drug delivery strategies that specifically target the tumor immune microenvironment, developing novel SIRPα-targeting therapeutics, and elucidating the molecular mechanisms of other SIRP family members. These efforts are crucial for advancing the clinical translation of SIRPα-targeted therapies for solid tumors. Further studies are warranted to dissect the cell type-specific functions of SIRPα across immune subsets and tumor cells, which will inform the development of precision immunotherapies tailored to distinct immunological and oncogenic contexts. Consequently, when designing therapeutic strategies targeting SIRPα-overexpressing cancers, it is critical to consider not only the immunostimulatory effects of SIRPα inhibition on myeloid cell-mediated immunity within the tumor microenvironment but also its direct impact on tumor cells and whether such effects may counteract potential immunotherapeutic benefits. In brief, targeting SIRPα may constitute a prospective path for future research in cancer immunotherapy, and studying the role of endogenous SIRPα in cancer cells and progression has significant scientific value.
